# MARCO^+^ Macrophage Dynamics in Regenerating Liver after 70% Liver Resection in Mice

**DOI:** 10.3390/biomedicines9091129

**Published:** 2021-09-01

**Authors:** Andrey Elchaninov, Anastasia Lokhonina, Polina Vishnyakova, Anna Soboleva, Anastasiya Poltavets, Daria Artemova, Andrey Makarov, Valeria Glinkina, Dmitry Goldshtein, Galina Bolshakova, Gennady Sukhikh, Timur Fatkhudinov

**Affiliations:** 1 Laboratory of Regenerative Medicine, National Medical Research Center for Obstetrics, Gynecology and Perinatology Named after Academician V.I. Kulakov of Ministry of Healthcare of Russian Federation, 117997 Moscow, Russia; nastya.serbsky@gmail.com (A.L.); vpa2002@mail.ru (P.V.); a.s.poltavets@gmail.com (A.P.); g_sukhikh@oparina4.ru (G.S.); 2 Histology Department, Medical Institute, Peoples’ Friendship University of Russia (RUDN University), 117198 Moscow, Russia; anvitmak@yandex.ru (A.M.); tfat@yandex.ru (T.F.); 3 Laboratory of Growth and Development, Scientific Research Institute of Human Morphology, 117418 Moscow, Russia; annasobo@mail.ru (A.S.); artiomova.darya@yandex.ru (D.A.); gbolshakova@gmail.com (G.B.); 4 Histology Department, Pirogov Russian National Research Medical University, Ministry of Healthcare of the Russian Federation, 117997 Moscow, Russia; vglinkina@mail.ru; 5 Stem Cell Genetics Laboratory, Research Centre for Medical Genetics, 115522 Moscow, Russia; dv@rm7.ru

**Keywords:** macrophages, Kupffer cells, liver, regeneration, MARCO, TIM4

## Abstract

Background: Macrophages play a key role in liver regeneration. The fates of resident macrophages after 70% resection are poorly investigated. In this work, using the MARCO macrophage marker (abbreviated from macrophage receptor with collagenous structure), we studied the dynamics of mouse liver resident macrophages after 70% resection. Methods: In BALB/c male mice, a model of liver regeneration after 70% resection was reproduced. The dynamics of markers CD68, TIM4, and MARCO were studied immunohistochemically and by using a Western blot. Results: The number of MARCO- and CD68-positive macrophages in the regenerating liver increased 1 day and 3 days after resection, respectively. At the same time, the content of the MARCO protein increased in the sorted macrophages of the regenerating liver on the third day. Conclusions: The data indicate that the number of MARCO-positive macrophages in the regenerating liver increases due to the activation of MARCO synthesis in the liver macrophages. The increased expression of MARCO by macrophages can be regarded as a sign of their activation. In the present study, stimulation with LPS led to an increase in the expression of the *Marco* gene in both Kupffer cells and macrophages of bone marrow origin.

## 1. Introduction

The liver contains the largest population of macrophages in the mammalian body [1]. Resident liver macrophages, also known as Kupffer cells, are integral participants of various processes in the liver under both normal and pathological conditions [2]. Notwithstanding the natural abundance of Kupffer cells, acute hepatotoxicity causes colonization of the liver by high numbers of blood monocytes [3,4]. This phenomenon may indicate divergent functions of bone marrow-derived monocytic macrophages and Kupffer cells [5,6], which descend from erythro-myeloid progenitors of the yolk sac wall [7,8].

Repair processes in the liver after acute hepatotoxic injury are fairly well described in scientific literature, and a number of good experimental models for this condition have been successfully developed [9]. Completely different regeneration patterns, however, arise after a massive loss in hepatic tissue volume, complying to a different classical experimental model of 70% liver resection (hepatectomy) in laboratory rodents [10]. Despite the widespread use of hepatectomy models, the extent (and even the very fact) of monocyte/macrophage migration to the liver remnant after massive resections remains questionable [11,12]. The controversy of the existing evidence is apparently related to species-specific differences in the rates of resection-induced macrophage immigration. Moreover, resection volume per se (which differs considerably between the studies) may represent a critical variable linked to the extent of macrophage immigration. Our own experiments demonstrated pronounced immigration of Ly6C^+^ monocytes to the liver after 70% hepatectomy in mice. The immigration was accompanied by proliferation and a modest increase in cell death rates of macrophages within the liver, although ascribing these dynamics to a particular subtype of liver macrophages (migratory or resident) was problematic [13].

A number of studies performed on the acute hepatotoxicity models indicate a substantial loss of Kupffer cells at the early stages of the recovery, neutralized by their subsequent compensatory proliferation [3,4]. For hepatectomy models, the corresponding dynamics of Kupffer cells is still obscure, not least due to the difficulties of selective tracking down of the resident liver macrophages as opposed to their monocytic counterparts, especially given the renowned high plasticity of macrophages and the limited specificity of available markers.

MARCO (abbreviated from macrophage receptor with collagenous structure), a scavenger receptor class A protein expressed on macrophages [14,15], has been featured as a liver mature macrophage-specific marker [16,17]. In this study, we estimate the dynamics of MARCO^+^ macrophages in the liver after 70% hepatectomy in mice. There are currently no data on changes in MARCO expression in liver macrophages during liver regeneration. At the same time, MARCO is considered as a possible marker of the M1 phenotype of macrophages [18]. Thus, the study of its expression will make it possible to clarify the direction of polarization of resident macrophages during liver regeneration. In this study, we estimate the dynamics of MARCO^+^ macrophages in the liver after 70% hepatectomy in mice.

## 2. Materials and Methods

### 2.1. Mice

BALB/c mice (males, body weight 20–22 g) obtained from the Stolbovaya branch of the Scientific Center for Biomedical Technologies of the Federal Medical and Biological Agency (Stolbovaya, the Moscow region, Russia) were housed in plastic cages at 22 ± 1 °C with a 6:00 a.m. to 6:00 p.m. illumination cycle and free access to water and food. The keeping and all procedures with the animals were carried out according to the International Recommendations for Conducting Biomedical Research Using Animals (1985) and corresponding regulations for laboratory practice in the Russian Federation (Order of the Ministry of Health of the Russian Federation No. 267, 2003, and federal law “On the protection of animals from abuse”, Ch. V, # 10, 4679-GD, 1999).

### 2.2. Animal Model

All manipulations with animals (*n* = 132) were carried out at 10–11 a.m. under general anesthesia with isoflurane. Partial hepatectomies (resections of 70% liver volume) were carried out by the conventional Higgins and Anderson procedure [19], with two groups of comparison, of 25 intact and 27 sham-operated animals, respectively. The operated animals, housed as previously described [13], were sacrificed in a CO_2_-chamber at 0 h, 24 h, 72 h, or 7 days, at least five mice of each group for a timepoint. Cell culture experiments were carried out on pools of sorted cells collected from five animals. The study was conducted according to the guidelines of the Declaration of Helsinki and approved by the Institutional Review at the Scientific Research Institute of Human Morphology on 1 February 2019 (Protocol No. 3).

### 2.3. Isolation of Macrophages

The livers were perfused through vena portae with phosphate buffered saline (PBS). Stromal elements were eliminated by enzymatic digestion with collagenases I and IV (PanEco, Moscow, Russia). The collected total cells, suspended in saline to a 30 mL final volume, were precipitated for 3 min at 50 g. The pellet (comprising hepatocytes) was discarded, and the supernatant was collected for subsequent gradient centrifugation on Lympholyte-M (Cedarlane, Burlington, ON, Canada) yielding a fraction comprising macrophages. This fraction was subjected to immunomagnetic sorting on a MidiMACS™ Separator using the Anti-F4/80 MicroBeads UltraPure magnetic beads-filled LS Columns (Miltenyi Biotec, Bergisch Gladbach, Germany) according to the manual.

Pure cultures of bone marrow-derived macrophages were obtained from monocytes of peripheral blood. Collected blood samples were mixed with an equal volume of Hanks’ balanced salt solution (HBSS) containing 1000 ME/mL heparin (Sintez, Kurgan, Russia). The fraction of mononuclear cells was isolated by gradient centrifugation (400× *g* for 30 min at 20 °C) on Ficoll (PanEco, Moscow, Russia) and washed twice with HBSS (300× *g*, 20 min, 20 °C). Viable cells were quantitated using a TC20 automated cell counter (Bio-Rad, Hercules, CA, USA) and sorted immunomagnetically using the MidiMACS™ Separator with the Anti-CD115 (Monocyte Isolation Kit (BM), mouse) magnetic beads-filled LS Columns (Miltenyi Biotec, Bergisch Gladbach, Germany) according to the manual. To obtain cultures with stable mature macrophage phenotypes, the obtained monocytes were incubated for 24 h with MCSF. Each culture represented pooled samples from five animals.

### 2.4. Flow Cytometry Assay

The purity of all isolated macrophage populations was assessed by flow cytometry with antibodies to either F4/80 (anti-F4/80-PerCP-Vio700, anti-mouse, 1:100; Isotype control: REA Control-PerCP-Vio700, Miltenyi Biotec, Bergisch Gladbach, Germany) for Kupffer cells or CD115 for monocytes (anti-CD115, FITC, anti-mouse, 1:100; Isotype control: human IgG1, FITC, REAfinity™).

Each sample consisted of pooled sorted cells from five animals. Immunostaining for intracellular markers was carried out using the Inside Stain Kit (Miltenyi Biotec, Bergisch Gladbach, Germany). Immunophenotyping for surface markers involved exposure of the cells (1 × 10^5^ in 100 μL PBS) to specific antibodies and corresponding serotype controls. The analysis was performed on a Cytomics FC 500 flow cytometer (Beckman Coulter, Brea, CA, USA) with the CXP software (Beckman Coulter, Brea, CA, USA). The cells were gated by forward and side scattering to define the region of interest and exclude debris.

Immunophenotyping was accomplished similarly by gating the main population of sorted cells after staining for surface markers CD115 and F4/80. The percentages of immunonegative cells were determined in relation to isotype controls performed for each sample, with the quadrant axes in dot plots set accordingly. In all obtained samples of monocytes and Kupferr cells, the proportion of CD115 or F4/80 positive cells, respectively, was above 90% (Supplementary Material, Figure S1).

### 2.5. Cell Culture of Macrophages

To obtain pure cultures, the macrophages from liver tissues and the monocytes from peripheral blood were treated similarly. The cells were maintained in an RPMI medium (PanEco, Moscow, Russia) with 10% fetal calf serum (PAA Lab, Pasching, Oberösterreich, Austria), 1% penicillin-streptomycin (PanEco, Moscow, Russia), and 50 ng/mL MCSF (Miltenyi Biotec, Bergisch Gladbach, Germany). The growth medium was replaced with a fresh portion on day 2, and all non-attached cells were discarded.

### 2.6. Activation of Macrophage Cell Cultures with LPS

The activation of Kupffer cells and monocytic macrophages in pure cultures was carried out by applying 50 ng/mL LPS (Sigma-Aldrich, St. Louis, MO, USA) in an RPMI medium (PanEco, Moscow, Russia), supplemented with 10% fetal calf serum and 1% penicillin-streptomycin, for 24 h.

### 2.7. Immunocytochemistry and Immunohistochemistry

For immunocytochemistry, macrophages were cultured on coverslips and fixed with 4% paraformaldehyde in PBS. Immunohistochemistry was performed on hepatic tissues preserved in liquid nitrogen and cryosectioned at 5–7 µm. For immunostaining, the sections (or coverslips with attached cells) were exposed to combinations of anti-Vimentin (1:100, Abcam, Cambridge, UK), anti-CD68 (1:100, Abcam, UK) with PE-conjugated secondary antibodies (1:200, Santa Cruz Biotechnology, Santa Cruz, CA, USA) or anti-MARCO (1:100, Abcam, Cambridge, UK) with FITC-conjugated secondary antibodies (1:200, Abcam, Cambridge, UK), and the nuclei were counterstained with 4′,6-diamidino-2-phenylindole (DAPI, Sigma-Aldrich, St. Louis, MO, USA). Positivity indexes were determined as proportions of immunopositive cells in total counts.

### 2.8. Reverse Transcription Polymerase Chain Reaction (RT PCR) Gene Expression Assay

Total RNA, isolated from the cells or liver tissues with the RNeasy Plus Mini Kit (Qiagen, Hilden, Germany), was used for the random-primed cDNA synthesis with MMLV RT kit (Evrogen, Moscow, Russia). PCRs were run in duplicates based on the qPCR mix-HS SYBR 5x master mix (Evrogen, Moscow, Russia) using transcript-specific primers (listed in Table 1). The expression was quantified by the threshold cycle (Ct) method, with relative expression levels calculated against *Gapdh* [20].

### 2.9. Immunoblotting

Total proteins isolated from cells or tissues were separated by electrophoresis and transferred to blotting membranes as described elsewhere [5,13]. The membranes were preblocked, incubated overnight with antibodies to MARCO, TIM4, CD68, or GAPDH (anti-MARCO—ab 256822, 1:200; anti-TIM4—ab47637, anti-CD68-ab ab125212, anti-GAPDH—sc-25778, Abcam, Cambridge, UK), washed, and incubated with HRP-conjugated antibodies (Bio-Rad, Hercules, CA, USA). The bands were developed with Novex ECL Kit (Thermo Fisher, Waltham, MA, USA) in a ChemiDoc™ system (Bio-Rad, Hercules, CA, USA). The densitometry was carried out using ImageLab Software (Bio-Rad, Hercules, CA, USA) against GAPDH as a reference protein. For an image of the uncropped membrane after blotting, see Supplementary Material (Figure S2).

### 2.10. Statistics

The SigmaStat 3.5 program package (Systat Software Inc., San Jose, CA, USA) was used for the analysis. The Mann–Whitney U test was applied to evaluate the observed differences in gene expression. One-way ANOVA with the post hoc Holm-Sidak test or ANOVA on Ranks with the post hoc Tukey test or Dunn’s Method were applied for more-than-two-groups comparisons. All comparisons were made at a 0.05 level of significance.

## 3. Results

### 3.1. Macrophage Population Dynamics in Regenerating Livers

**Figure 1:**
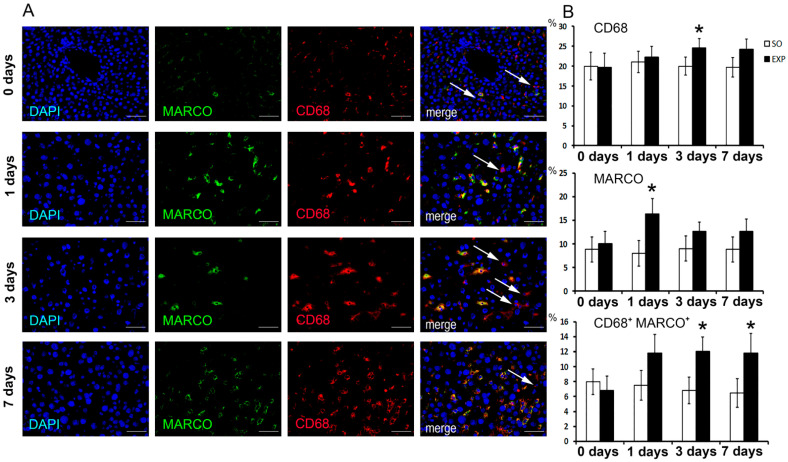
Immunohistochemical assessment of CD68 and MARCO expression in hepatic tissues after 70% liver resection in mice. (**A**) Fluorescent microscopy images displaying anti-CD68 (PE, red) and anti-MARCO (FITC, green) immunostaining and the counterstained cell nuclei (DAPI, blue), magnification ×400; bars, 50 µm. Arrows indicate MARCO-negative CD68^+^ macrophages. Percentage values of positively stained cells are given in the plots. (**B**) The data are presented as means ± standard deviations, with at least five specimens studied for each time point after the resection. EXP—hepatectomized mice, SO—sham-operated mice, * —statistical significance of the differences with 0 h time point (*p* < 0.05).

At 24 h (“day 1”) after the resection, MARCO^+^ macrophages significantly increased in number compared with intact and sham-operated controls (*p* < 0.05). On day 3 after the resection, CD68^+^ as well as double-positive CD68^+^MARCO^+^ macrophages significantly increased in number compared with intact and sham-operated controls (*p* < 0.05). The differences were evened out later on, as the regeneration proceeded (Figure 1A,B). Noteworthily, MARCO^+^ cell numbers were always lower than CD68^+^ cell numbers, consistently with the assumptions on marker specificity (Figure 1A,B).

### 3.2. Transcriptional Dynamics of Macrophage Marker-Encoding Genes in Regenerating Livers

**Figure 2:**
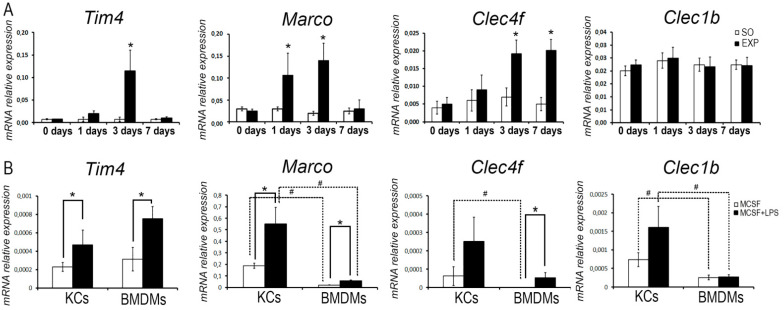
Expression of macrophage marker genes in regenerating livers (**A**) and in macrophages under the influence of LPS (**B**). (**A**). Expression of macrophage marker genes in regenerating liver studied by RT-PCR. (**B**) Expression of *Marco*, *Tim4*, *Clec4f*, and *Clec1b* genes studied by RT-PCR in bone marrow-derived macrophages (BMDMs) and Kupffer cells (KCs). The data are presented as means ± standard deviations, with at least five specimens studied for each time-point after the resection. EXP—hepatectomized mice, SO—sham-operated mice, *, # —the statistical significance of the differences with sham-operated control or corresponding group (*p* < 0.05).

Elevated mRNA levels in hepatic tissues were revealed for *Tim4* on day 3, for *Marco* on days 1 and 3, and for *Clec4f* on days 3 and 7 after the resection. For *Clec1b*, no significant changes in expression were observed in the experiments (Figure 2A).

### 3.3. Comparative Analysis of Lipopolysaccharide (LPS) Effects on the Expression of Macrophage Markers in Bone Marrow-Derived Macrophages and Kupffer Cells

LPS administration enhanced the expression of *Tim4* and *Marco* in pure cultures for both bone marrow-derived monocytic macrophages and Kupffer cells (*p* < 0.05). It should be noted that Kupffer cells initially expressed *Marco* and *Clec4f* at higher levels (*p* < 0.05) and this pattern was preserved upon LPS administration (Figure 2B). The LPS-induced enhancement of *Clec4f* expression was observed in monocytic macrophages only (*p* < 0.05; Figure 2B). The expression of *Clec1b* was also initially higher in Kupffer cells compared with monocytic macrophages, and remained such under the LPS stimulation, while no LPS-induced enhancement of *Clec1b* expression was observed for either subtype of macrophages (*p* < 0.05; Figure 2B).

**Figure 3:**
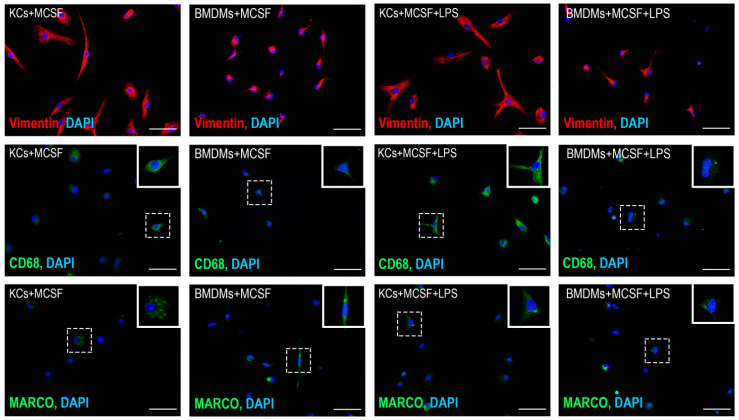
Immunofluorescence detection of Vimentin, CD68, and MARCO proteins in cultured bone marrow-derived macrophages (BMDMs) and Kupffer cells (KCs). Fluorescent microscopy images displaying anti-Vimentin and anti-CD68 (PE, red), anti-MARCO (FITC, green) immunostaining, and the counterstained cell nuclei (DAPI, blue), magnification ×400; bars, 50 µm. The highlighted dotted line cells are shown in the enlarged boxes.

Under the conditions of cultivation, macrophages obtained from blood monocytes acquired a process shape, which is clearly seen from the expression of vimentin. Kupffer’s cells initially had a large number of processes, which were preserved under cultivation conditions (Figure 3). Upon visual assessment of the expression of CD68, it can be noted that the expression of this marker was higher in Kupffer cells compared to macrophages of bone marrow origin, both under conditions of cultivation with MCSF and under the influence of LPS (Figure 3). When assessing the expression of the MARCO marker, it is worth noting the heterogeneity of macrophages for this trait, which was retained under the influence of LPS (Figure 3).

### 3.4. Protein Expression Dynamics for CD68, TIM4, and MARCO in Total Tissues and Macrophages of Regenerating Livers

**Figure 4:**
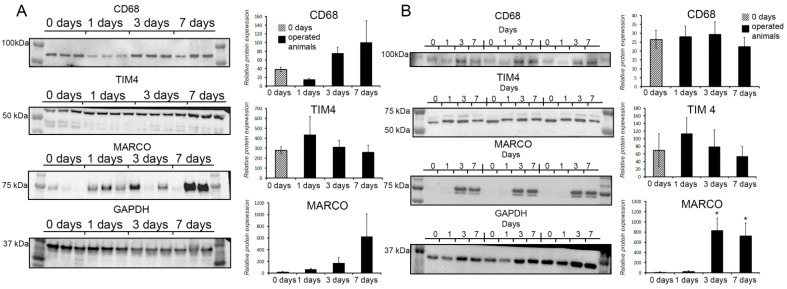
Evaluation of the content CD68, TIM4, and MARCO proteins in tissues (**A**) and isolated macrophages of regenerating liver (**B**) by immunoblotting. Representative images of the signals are shown in panel C, complemented with densitometry; full-size membrane images are given in Supplementary Material Figure S2. The data are presented as means ± standard deviations, with at least five specimens studied for each time point after the resection. *—statistical significance of the differences with 0 h time point (*p* < 0.05).

No changes in the content of TIM4 protein in total hepatic tissues, or macrophages isolated from them, were observed in the experiments (Figure 4A,B).

The dynamics for the CD68 protein were subtle. According to ANOVA on Ranks, the CD68 protein content in regenerating livers was significantly altered compared with the controls (*p* < 0.05), whereas a post hoc pairwise comparison by the Tukey Test indicated the lack of significance. In this regard, we can only note a downward trend most evident at the 24 h time-point (Figure 4A).

The dynamics for the MARCO protein was subtle as well; a certain upward trend around day 7, albeit below significance, should be noted (Figure 4A). However, macrophages isolated from hepatic tissues expressed higher quantities of the MARCO protein on days 3 and 7 after the resection compared with the control isolates (*p* < 0.05; Figure 4B).

## 4. Discussion

Regeneration capacity is shared to a variable extent by all living organisms [21]. Repair processes in mammals follow certain patterns (including both advantages and limitations) not observed in other multicellular animals. One of the characteristic regenerative traits in mammals is the pronounced regenerative capacity of parenchymal organs [22,23]. Among these, the liver is distinguished by the highest regenerative potential, which makes it a constant research focus. The restoration of liver mass after 70% resection in laboratory rodents represents a classic model of regenerative biology and medicine [2,10].

In terrestrial vertebrates, repair processes are largely orchestrated by the immune system. The site of injury is typically subject to massive infiltration with leukocytes, notably monocytes and macrophages [24]. In the liver, under physiological conditions, numerous populations of immunocompetent cells are found, among which, in addition to macrophages (20% of non-parenchymal cells), NK cells (6% of non-parenchymal cells) and NKT cells (8% of non-parenchymal cells) are found, for other types of leukocytes (eosinophils, neutrophils, lymphocytes) account for approximately 10% of all non-parenchymal cells [25,26]. The role of these immunocompetent cells is extremely diverse both in normal conditions since the liver is a place for the utilization of a large amount of harmful metabolic products, xenobiotics, endotoxin, and during reparative processes. Despite the large population of immunocompetent cells in the liver, infiltration with leukocytes can be observed in the liver under the conditions of acute hepatotoxic injury [3,4]. However, the restoration of liver mass after ‘clean’ resections stands apart in this regard. The lack of pronounced inflammatory cell infiltration in remnant livers has been described in several studies [27,28]. However, with the advance of methods for the observation and detection of infiltrating immune cells, eventually evidence accumulated confirming the migration of certain cells to the liver after partial hepatectomy, notably eosinophils [29], macrophages and monocytes [12,13], and also neutrophils [30]. Thus, despite the less conspicuous character of infiltration, the model of liver regeneration after resections in laboratory animals is generally consistent with other cases of tissue repair in vertebrates in terms of definite involvement of the immune system.

Migration of monocytes and other leukocytes to the remnant liver is consistent with the generic pattern of tissue repair in mammals, thus adding to the feasibility of using partial hepatectomy in rodents as a universal model. On the other hand, a new question arises in connection with the aforementioned features of hepatic tissue and its cellular composition. What is the reason for an organ with the highest density of macrophages (the intact liver harbors over 90% of all macrophages of the body) to additionally accept a huge number of immigrating monocytes during regeneration? As we have already noted, the situation may reflect divergent roles of the resident Kupffer cells and the immigrating bone marrow-derived macrophages: The former are involved predominantly in the maintenance of liver homeostasis, whereas demand for the latter is invariably concerned with repair [5,6].

Priming of hepatocytes by a certain combination of proliferative signals is of primary importance for successful liver repair [27,28]. The action of TNFα and IL6 on hepatocytes is indispensable for the priming [31]. The role of Kupffer cells as the principal source of TNFα and IL6 in remnant livers has been demonstrated by a number of studies [27,28]. TNFα in damaged tissues is produced mainly by the so-called non-classical monocytes (Ly6C^low^ monocytes in mice and CD16^+^ monocytes in humans), which migrate to the site of injury in advance of the classical monocytes [32,33]. Under conditions of acute hepatotoxic injury, early phases of the recovery are marked by massive death of Kupffer cells [3,4]; the coinciding massive arrival of blood monocytes may be therefore considered as a compensatory event.

The rates of cell death for Kupffer cells after partial hepatectomy in rodents remain unspecified. Our own data indicate an increase in cell death rates of F4/80^+^ liver macrophages at 24 h after 70% liver resection in mice [13]. Specific contributions of the resident Kupffer cells and the arriving monocytic macrophages to this dynamic remain implicit, due to the lack of reliable immunochemical targets to make a distinction between the two subtypes. The destiny of monocytes/macrophages that arrive in the remnant liver thus remains disputable. Some studies show that these cells are highly transient and die, along with the recovery of the resident macrophage population [3,4]. Other studies demonstrate (re)differentiation of the newly arrived monocytic macrophages into phenotype(s) identical to Kupffer cells [16,17].

Here we study macrophage dynamics in the same murine model by adding the MARCO protein, a recently characterized cell surface marker of mature macrophages, as a target for immunostaining.

MARCO is expressed on subsets of macrophages and dendritic cells; it upregulates on macrophages after bacterial infection, and an important role for this bacteria-binding molecule is suggested in the removal of pathogens. M1 macrophages are classically activated, typically by lipopolysaccharide (LPS), and produce proinflammatory cytokines, phagocytize microbes. Few studies showed that the MARCO expression after LPS induction increased [34] and the fact that structural resemblance of the MARCO molecule to the scavenger receptor proves that the bacterial cell walls and related components are ligands for it too [35]. So, MARCO could be used as an M1 marker [36]. Since MARCO is expressed on both macrophages and dendritic cells, it is likely the link between innate and adaptive immunity. The relationship between MARCO and TLR-induced inflammation has been established. MARCO, like other class A scavenger receptors, quickly binds pathogens and internalizes them, which prevents ligands from binding to surface TLRs, while increasing their recognition by intracellular sensors [37]. Tumor-associated macrophages (TAMs) conventionally classified as M2 [38] are a heterogeneous population. Some TAMs could switch their phenotype in the influence of the tumor microenvironment. Additional studies that will prove the fact that MARCO^+^ TAMs are M2 polarized should be conducted.

At early time-points after the resection, a decrease in MARCO^+^ cell counts would be expectable, reflecting both the increased cell death rates tendency and the increase in the proportion of MARCO^−^ young monocytic macrophages; a similar dynamic of MARCO content in hepatic tissue would have been revealed by Western blot. Contrary to these predictions, we observed an increase in the proportion of MARCO^+^ macrophages at 24 h after the resection (as assessed by immunohistochemistry) and an increase in the MARCO protein content of F4/80^+^ macrophages isolated from regenerating livers by magnetic sorting on days 3 and 7 (as assessed by Western blotting). These findings suggest that the resection leads to activation of MARCO synthesis not only in the migratory macrophages of bone marrow origin but also in the resident macrophages of the liver (Kupffer cells).

Specific contributions of these macrophage subpopulations to the observed increase in MARCO protein content of the liver tissues during regeneration are unclear. It can be suggested that the arriving monocytic macrophages, which eventually mature, tend to express MARCO at higher levels compared with the resident Kupffer cells. This is the only likely explanation for the significantly elevated content of MARCO protein in macrophages isolated from hepatic tissues on day 7 after the resection when the regeneration process is virtually complete [39]. Comparative studies on MARCO expression in Kupffer cells (as opposed to monocytic macrophages of bone marrow origin) during the resection-induced liver regeneration are missing. Experimental re-colonization of the liver by bone marrow-derived macrophages after in vivo depletion of Kupffer cells indicates that the resident and the colonizing macrophage subtypes express MARCO at similar levels [16,17]. Here we demonstrate, however, that Kupffer cells cultured in the presence of macrophage colony-stimulating factor (MCSF) express *Marco* at higher levels than the bone marrow-derived monocytic macrophages cultured under the same conditions. At that, under the action of LPS, the expression of the *Marco* gene is increased approximately two-fold in bone marrow-derived monocytic macrophages and Kupffer cells similarly. Compiling these pieces of evidence, we suppose that the observed significant increase in MARCO protein content is due to the induction of its synthesis in resident macrophages of a regenerating liver.

Given the high level of MARCO expression in tissue macrophages, as well as in TAMs [38], it is possible to consider MARCO as a therapeutic target for the treatment of certain nosologies. In a non-small cell, lung cancer model, it was demonstrated that an accumulation of MARCO-expressing macrophages is associated with worse clinical outcomes and resistance to anticancer therapy [40,41]. Targeting MARCO-positive macrophages renews cytolytic activity and the antitumoral capacity of NK and T-cells and modulates the activity of regulatory T-cells. However, in the case of hepatocellular carcinoma, on the contrary, an increase in *Marco* expression led to tumor regression [42]. In experiments on an in vitro model of silica-induced inflammation, it was demonstrated that MARCO controls the fibrotic response through regulating the epithelial–mesenchymal transition and suggests a novel target for preventive intervention [43]. The downregulation of MARCO leads to susceptibility to postinfluenza bacterial pneumonia, a major cause of morbidity and mortality. Immunomodulation of MARCO could improve host defense and resistance to secondary bacterial pneumonia [44]. So, MARCO-positive macrophages can be considered as a therapeutic target not only in proliferative diseases [45], but also in infectious diseases (viral and bacterial) and neurodegenerative diseases such as Alzheimer’s disease [46].

TIM4 (T-cell immunoglobulin and mucin domain-containing molecule) is expressed on many cells of the monocytic-macrophage system, including dendritic cells and lymphocytes [47,48]. TIM4 recognizes phosphatidylserine-expressing apoptotic cells, nuclei of erythrocyte precursors, and, through interaction with integrins, participates in the removal of dying cells and their nuclei [49], as well as phosphatidylserine from exosomes, thus participating in the regulation of intercellular interactions. TIM4 blockade stimulates the maturation of T regulatory cells and improves allograft acceptance [50].

Despite the lack of significant alterations in TIM4 protein content, expression of the corresponding gene in hepatic tissues (and also in isolated liver macrophages) significantly increases after the resection, consistently with the LPS-induced elevated expression of *Tim4* in cultured liver macrophages observed by us in this study.

CLEC4F is a C-type lectin receptor that recognizes desialylated glycans and is considered a highly specific protein of liver macrophages [51]. The role of this receptor in the functioning of liver macrophages remains poorly understood. It is assumed that CLEC4F may be involved in the regulation of antigenic presentation of glycolipids by Kupffer cells [52], as well as in the removal of desialylated platelets [51].

CLEC1b (CLEC-2) is a C-type lectin-like receptor 2 that is expressed on B lymphocytes, granulocytes, monocytes, and dendritic cells, as well as Kupffer cells and platelets, and binds to various ligands including the mucin-like protein podoplanin (PDPN) [53]. It was found that LPS and other inducers of inflammation can increase the expression of CLEC-2, but this is not typical for all macrophage subpopulations [54]. According to some data, CLEC-2 in Kupffer cells is detected at a low level [53], while it can participate in the secretion of proinflammatory cytokines [55].

The LPS-induced increase of *Clec4f* expression in the cultures of bone marrow-derived monocytic macrophages, isolated from peripheral blood, should be noted as well. This effect apparently reflects the continued differentiation of monocytes into mature macrophage phenotypes.

Thus, the role of the considered receptors is somehow related to the regulation of inflammation and antigenic presentation. One of the possible triggers of the expression of the studied receptors is an increase in the level of LPS in the blood of hepatectomized animals. This is consistent with data demonstrating MARCO activation after bacterial infection [35]. The load on macrophages in the lobes remaining after resection increases not only for the utilization of LPS but also, for example, for the clearance of senescent erythrocytes and platelets [51], which probably leads to the activation of CLEC4F synthesis.

It should be mentioned that the counts of MARCO^+^ cells in our experiments were invariably lower than the counts of CD68^+^ cells for all animals independently of the resection status (true resection, sham resection, or intact). This situation may indicate heterogeneity of liver macrophages with regard to MARCO expression; also, elevated expression of CD68 in other cell types, except macrophages, cannot be excluded [56].

The dynamics of CD68^+^ cell counts and CD68 protein content of liver tissues after 70% hepatectomy in the studied model should be mentioned as well. CD68, a transmembrane glycoprotein, functions as a scavenger receptor (albeit of a different class than MARCO) heavily involved in phagocytosis [56]. The observed relative counts of CD68^+^ cells at 24 h after the resection may reflect the activation of phagocytosis rather than the pure increase in macrophage numbers. On the other hand, a decrease in CD68 content at the 24 h time-point was revealed in both the total hepatic tissues and the isolated liver macrophages by immunoblotting. This result may reflect the arrival of young macrophages, differentiating from migratory monocytes, which is consistent with previous studies by other authors [12].

## 5. Conclusions

In summary, enhanced synthesis of the macrophage protein MARCO has been observed in murine liver macrophages after 70% resection. The increased expression of MARCO by macrophages can be regarded as a sign of their activation, due to the established functional association of this protein with endocytosis [14,15]. The obtained results complement the existing data on the LPS-induced increase in expression of MARCO in bone marrow-derived macrophages [14,57]. The use of LPS stimulation in such models is biologically relevant, as hepatectomy is well-known to induce elevated blood levels of endotoxin (LPS), which contribute to liver macrophage activation and the onset of compensatory growth [58]. Apparently, the increase in relative counts of CD68^+^ cells in hepatic tissues after the resection is similarly indicative of liver macrophage activation, since the receptor glycoprotein CD68 also actively participates in endocytosis [56].

There are data in the literature indicating that during migration into the liver of monocytes and their further differentiation into macrophages, the expression of *Tim4*, *Marco*, *Clec1b*, and *Clec4f* increases [17]. As a result, “new” macrophages of bone marrow origin do not differ from resident macrophages of the liver. However, we found that the expression of the marker genes *Tim4*, *Marco*, *Clec1b*, and *Clec4f* probably also increases during liver resection in resident macrophages, which is probably associated with their activation and differentiation towards a phenotype adapted to regulate liver regeneration.

In the literature, *Marco* is considered as a marker of the M1 polarization of macrophages [18]. Thus, resident liver macrophages, upon regeneration after 70% resection, combine the high expression of the M1 phenotype marker MARCO, as well as M2 CD206 and CD163 [13]. Thus, the phenotype of liver macrophages during its regeneration cannot be unambiguously attributed to pro- or anti-inflammatory, which is consistent with the concept of the presence of a phenotypic continuum upon macrophage activation [18].

The clinical significance of the obtained data may lie in the fact that resident macrophages of the liver play a key role in the regulation of its homeostasis in normal conditions, during regeneration, and are also closely related to the development of such pathological processes as liver tumors and cirrhosis. Since after liver resection, a pronounced activation of Marco expression occurs, while organotypic regeneration of the parenchyma is observed without excessive deposition of collagen, the stimulation of MARCO synthesis in macrophages can likely be used as a possible target to stimulate the repair of the liver parenchyma and degradation of excessive deposits of the extracellular matrix and collagen in liver cirrhosis.

## Figures and Tables

**Table 1:** PCR primers for the mRNA target.

*Tim4*	for	ATTAACAACAAGTGAGTCTTTGCAG
	rev	CTTCAGTGTTGTCTGGCCTCT
*Marco*	for	CCAGGACTTTCAGGTGCCAA
	rev	TGGCCAGAAGACCCTTTCAT
*Clec4f*	for	TGCAGGACAGGCTTTAGGAA
	rev	TGACTTAGGCCCCAGTCCTTG
*Clec1b*	for	GTTGGATTGGATTATCACGCCAG
	rev	TCCCAGAAAGATTAATCCCGTT
*Gapdh*	for	AGGCCGGTGCTGAGTATGTC
	rev	TGCCTGCTTCACCACCTTCT

## Data Availability

The original contributions presented in the study are included in the article/supplementary files; further inquiries can be directed to the corresponding author/s.

## Supplementary Materials

The following are available online at https://www.mdpi.com/article/10.3390/biomedicines9091129/s1, Figure S1: Confirmation of purity of isolated cells by flow cytometry. Analysis of the number of CD 115 (A) and F4/80-positive cells (B). Representative forward, side scattering, and histogram are shown. The proportions of negative cells were determined in relation to the isotype controls. The percentages of positive cells are indicated. Figure S2: Full-size membrane after blotting of polyacrylamide gel. The Red arrow indicates the detected band of interest.
