# Supplementary material for: MARCO+ Macrophage Dynamics in Regenerating Liver after 70% Liver Resection in Mice
Source: Biomedicines. 2021 Sep 1;9(9):1129. doi: 10.3390/biomedicines9091129 (PMC8471044; doi:10.3390/biomedicines9091129)
Supplement: Supplementary file 1 [file biomedicines-09-01129-s001.zip › Fig1S.pdf]

Supplementary Material

MARCO+ MACROPHAGE DYNAMICS IN REGENERATING LIVER AFTER 70% LIVER RESECTION IN MICE

Andrey Elchaninov [1,2,*], Anastasia Lokhonina [1,2], Polina Vishnyakova [1,2], Anna Soboleva [3], Anastasiya Poltavets [1], Andrey Makarov [2] , Valeria Glinkina [4], Galina Bolshakova [3], Gennady Sukhikh [1] and Timur Fatkhudinov [2,3]

1 Laboratory of Regenerative Medicine, National Medical Research Center for Obstetrics, Gynecology and Perinatology Named after Academician V.I. Kulakov of Ministry of Healthcare of Russian Federation, 117997 Moscow, Russian Federation;

2 Histology Department, Medical Institute, Peoples' Friendship University of Russia (RUDN University), 117198, Moscow, Russian Federation

3 Laboratory of Growth and Development, Scientific Research Institute of Human Morphology, 117418 Moscow, Russian Federation;

4 Histology Department, Pirogov Russian National Research Medical University, Ministry of Healthcare of the Russian Federation, 117997, Moscow, Russian Federation

Correspondence: elchandrey@yandex.ru

Figure 1S. Confirmation of purity of isolated cells by flow cytometry. Analysis of the number of CD 115 (A) and F4 / 80-positive cells (B). Representative forward, side scattering and histogram are shown. The proportions of negative cells were determined in relation to the isotype controls. The percentages of positive cells are

indicated.

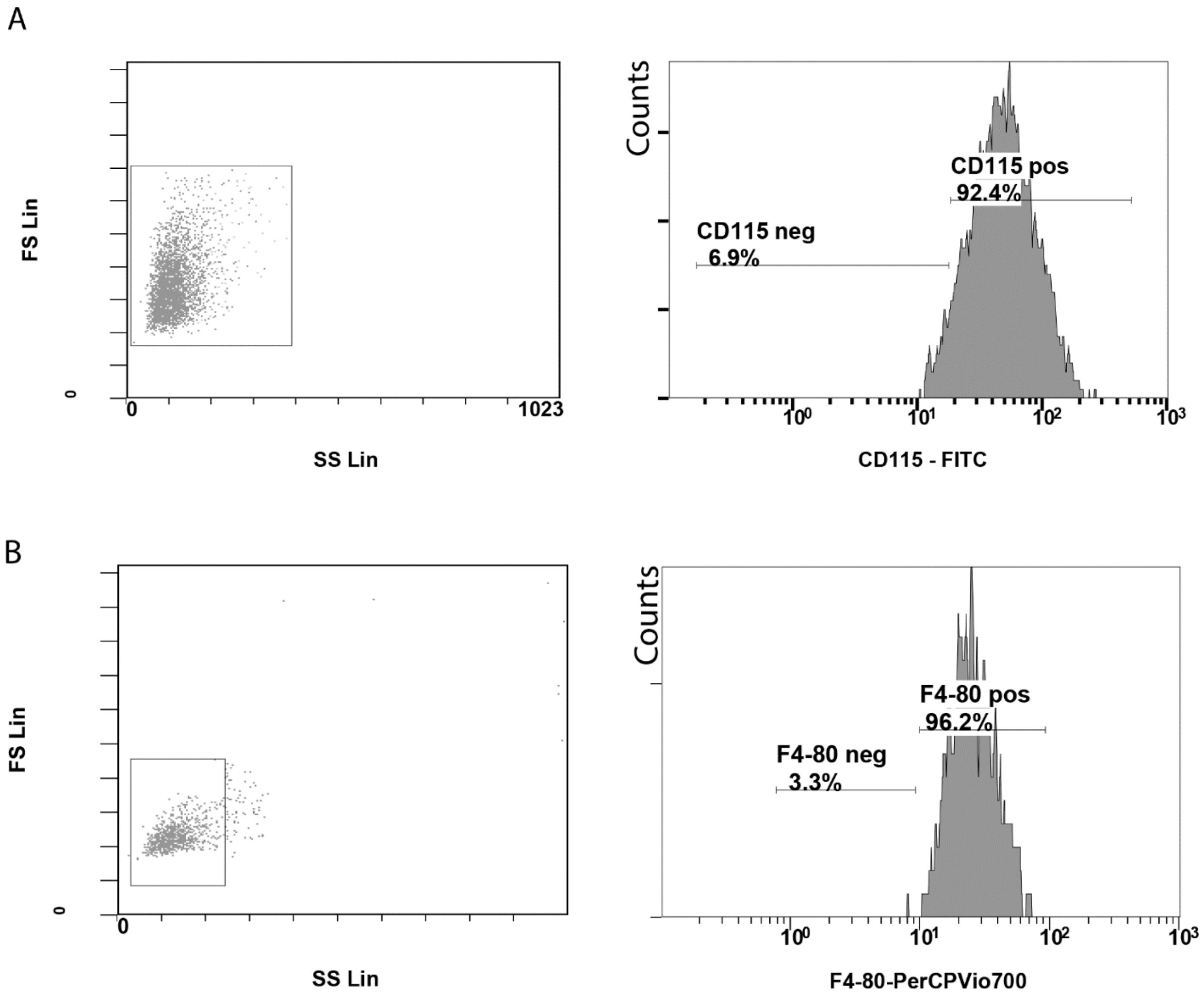
A
FS Lin
0
0
1023
SS Lin
Counts
CD115 pos
92.4%
CD115 neg
6.9%
10^0
10^1
10^2
10^3
CD115 - FITC
B
FS Lin
0
0
SS Lin
Counts
F4-80 pos
96.2%
F4-80 neg
3.3%
10^0
10^1
10^2
10^3
F4-80-PerCPVio700
