# Supplementary material for: MARCO+ Macrophage Dynamics in Regenerating Liver after 70% Liver Resection in Mice
Source: Biomedicines. 2021 Sep 1;9(9):1129. doi: 10.3390/biomedicines9091129 (PMC8471044; doi:10.3390/biomedicines9091129)

Supplementary Material

MARCO+ MACROPHAGE DYNAMICS IN REGENERATING LIVER AFTER 70% LIVER RESECTION IN MICE

Andrey Elchaninov [1,2,*], Anastasia Lokhonina [1,2], Polina Vishnyakova [1,2], Anna Soboleva [3], Anastasiya Poltavets [1], Andrey Makarov [2] , Valeria Glinkina [4], Galina Bolshakova [3], Gennady Sukhikh [1] and Timur Fatkhudinov [2,3]

1 Laboratory of Regenerative Medicine, National Medical Research Center for Obstetrics, Gynecology and Perinatology Named after Academician V.I. Kulakov of Ministry of Healthcare of Russian Federation, 117997 Moscow, Russian Federation;

2 Histology Department, Medical Institute, Peoples' Friendship University of Russia (RUDN University), 117198, Moscow, Russian Federation

3 Laboratory of Growth and Development, Scientific Research Institute of Human Morphology, 117418 Moscow, Russian Federation;

4 Histology Department, Pirogov Russian National Research Medical University, Ministry of Healthcare of the Russian Federation, 117997, Moscow, Russian Federation

- Correspondence: elchandrey@yandex.ru

Figure 2S. Full-size membrane after blotting of polyacrylamide gel. Red arrow indicates the detected band of interest.

Liver regeneration tissue

GAPDH

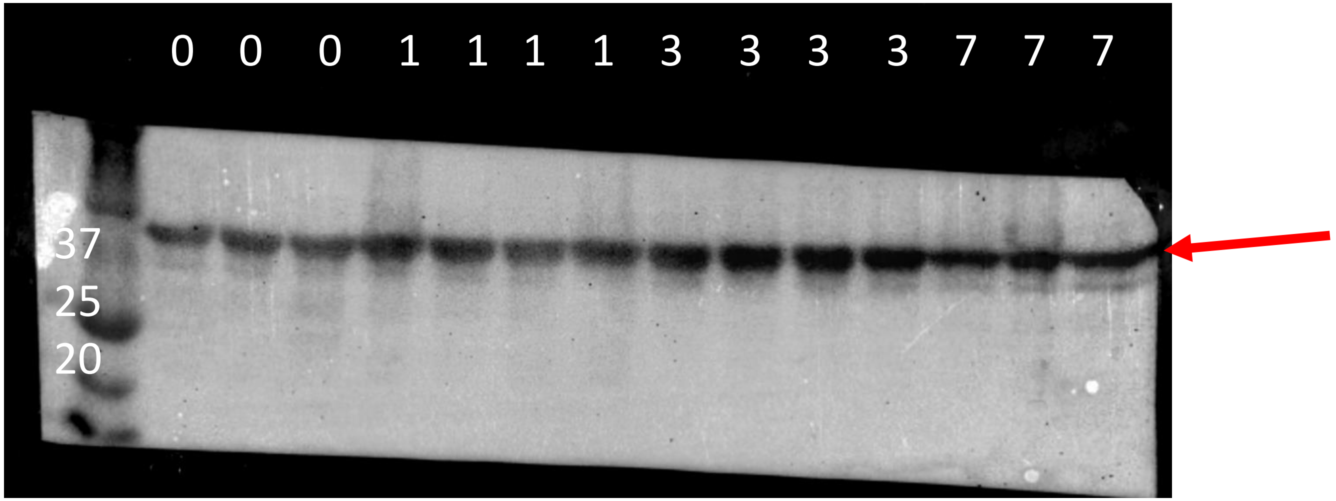

MARCO

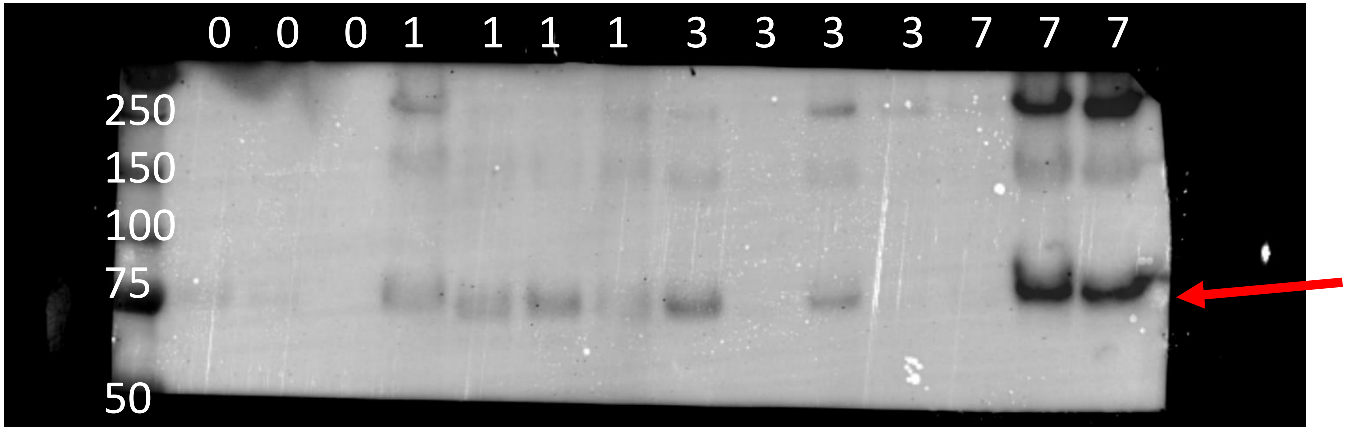

Liver regeneration tissue

TIM4

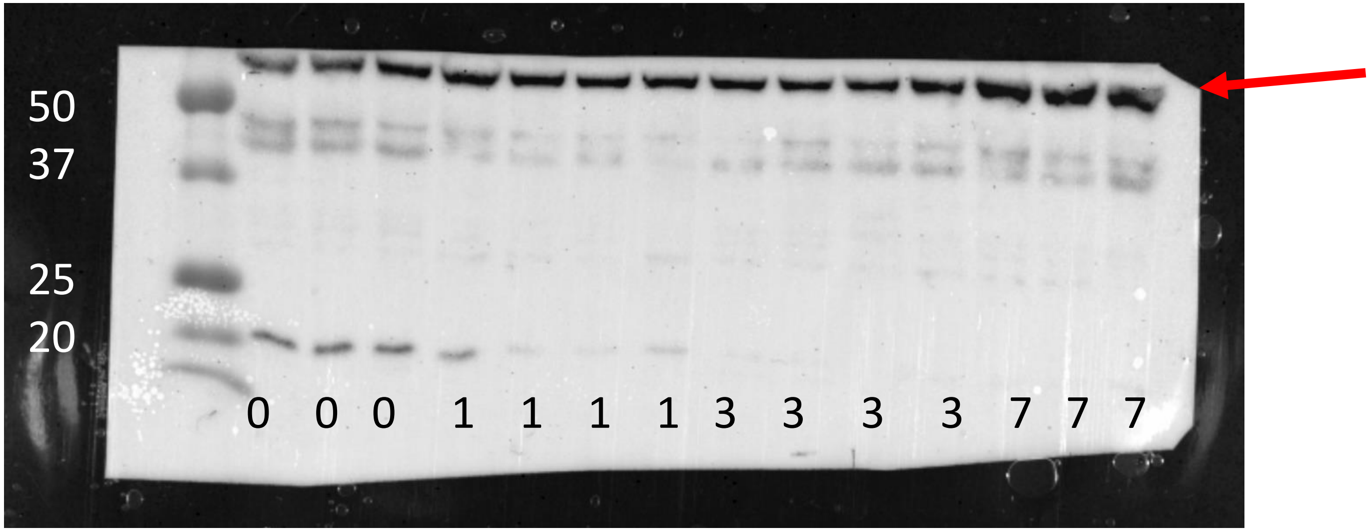

Liver regeneration tissue

CD68

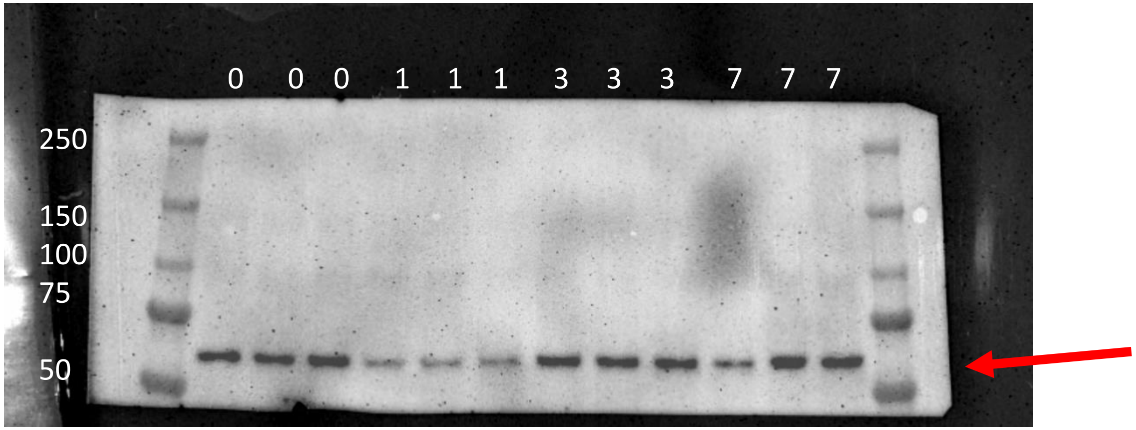

Liver regeneration tissue

GAPDH

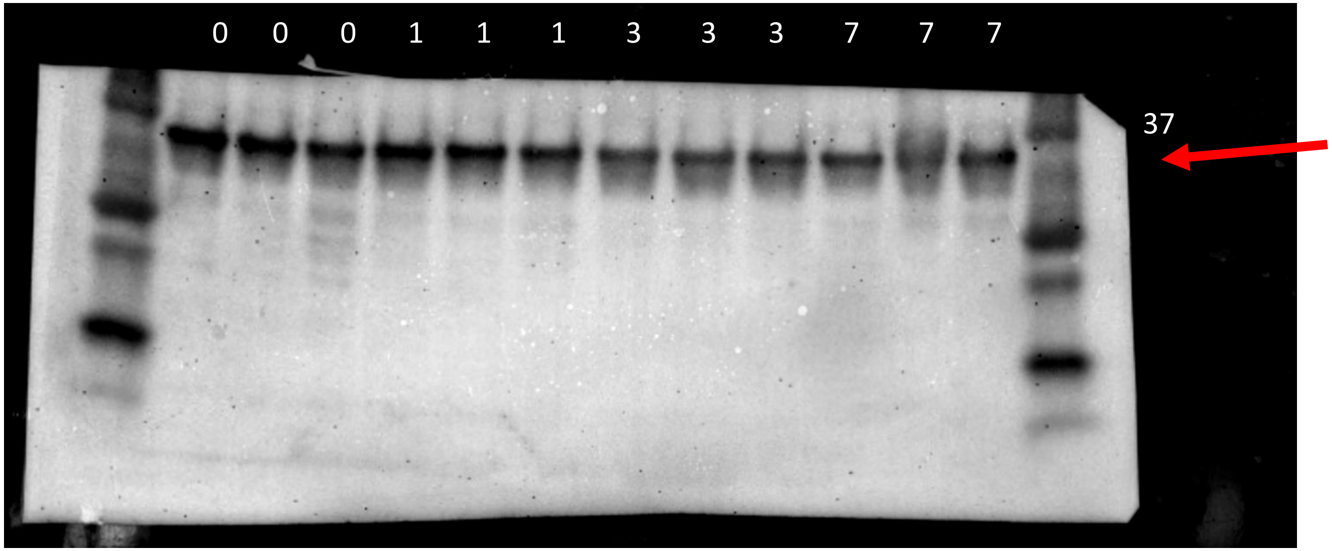

Liver regeneration tissue

MARCO

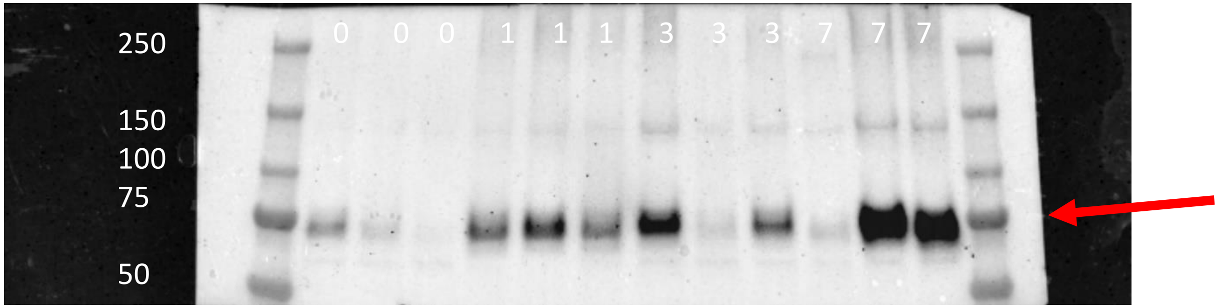

Sorted F4/80 liver macrophages

GAPDH

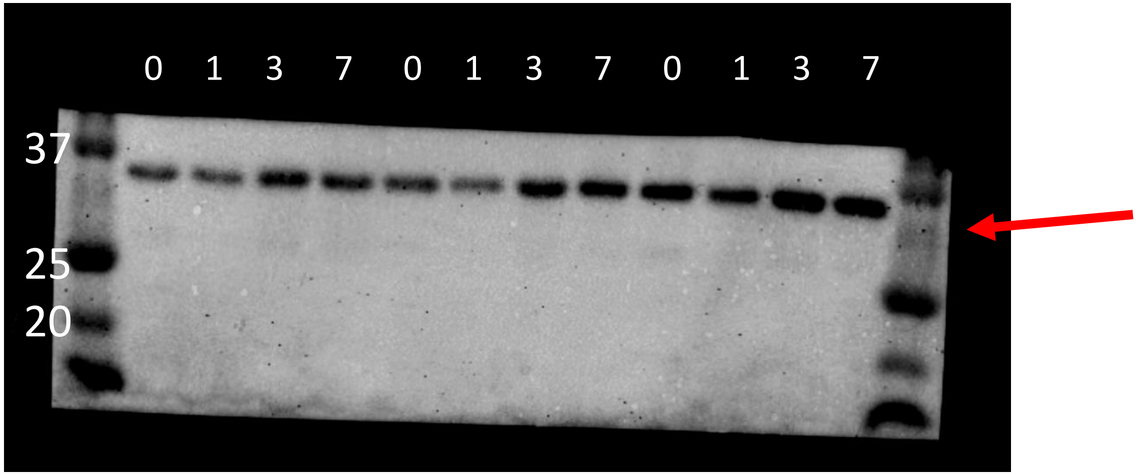

MARCO

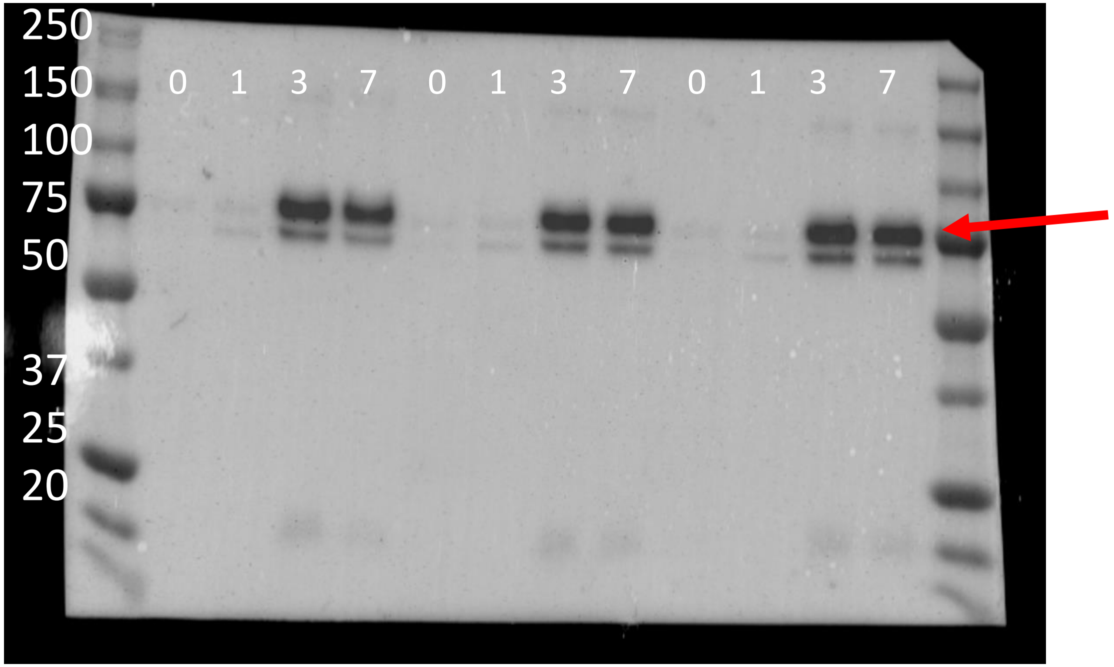

Sorted F4/80 liver macrophages

TIM4

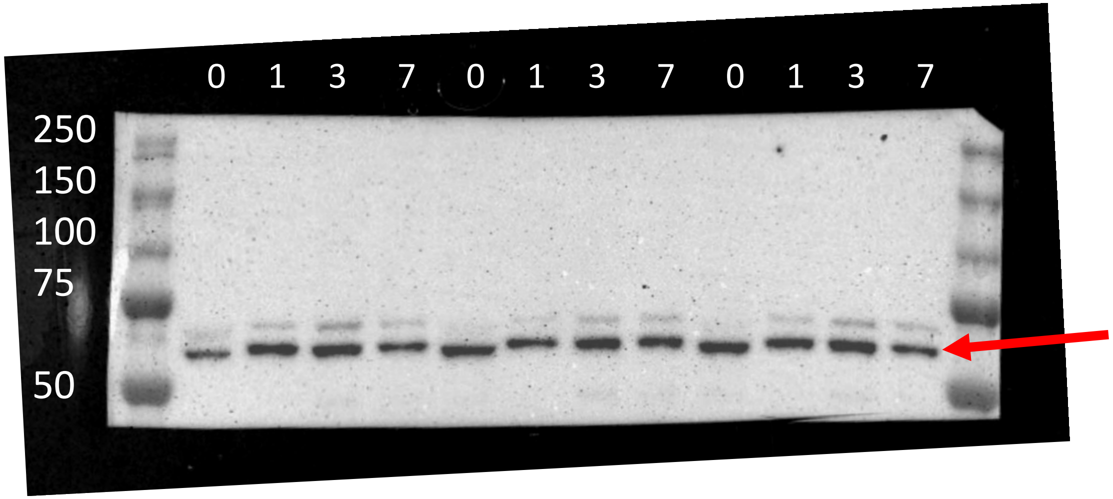

Sorted F4/80 liver macrophages

CD68

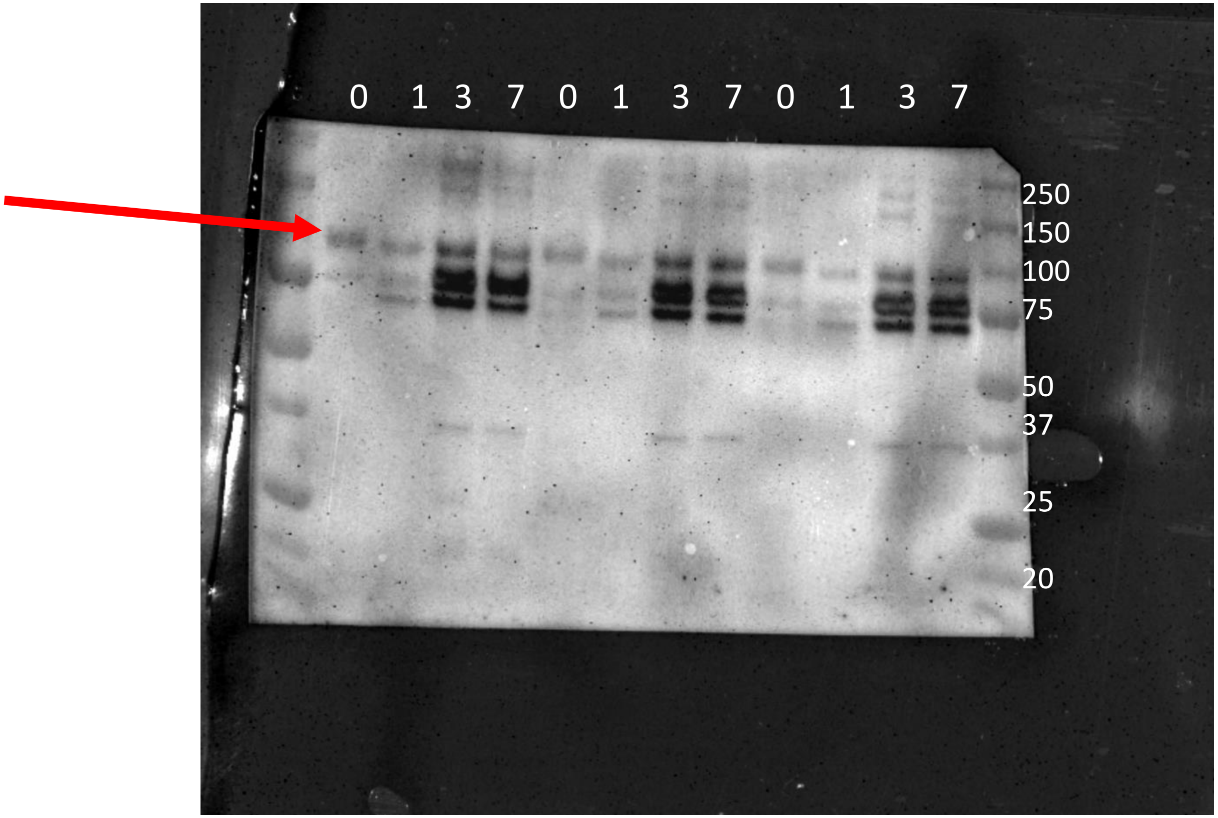

Supplement: Supplementary file 1 [file biomedicines-09-01129-s001.zip › Fig2S.pdf]
